# A New Comestible Formulation of Parasiticide Fungi to Reduce the Risk of Soil-Transmitted Helminth Infections in a Canine Shelter

**DOI:** 10.3390/pathogens11111391

**Published:** 2022-11-21

**Authors:** Cándido Viña, Rami Salmo, María Vilá Pena, Antonio Miguel Palomero, José Ángel Hernández, Cristiana Cazapal-Monteiro, María Sol Arias, Rita Sánchez-Andrade, Adolfo Paz-Silva

**Affiliations:** Control of Parasites Group (COPAR, GI-2120), Department of Animal Pathology, Faculty of Veterinary, University of Santiago de Compostela, 27002 Lugo, Spain

**Keywords:** dogs, endoparasites, prevention, soil filamentous fungi, edible

## Abstract

Dogs cared for in a shelter are dewormed every three–four months, but they all become infected one–two months later by the soil-transmitted helminths (STHs) *Toxocara canis*, *Toxascaris leonina*, *Trichuris vulpis,* and *Ancylostoma caninum*. For the purpose of reducing their risk of infection by decreasing the survival of helminths’ infective stages in soil, chlamydospores of two parasiticide fungi, *Mucor circinelloides* (ovicide) and *Duddingtonia flagrans* (larvicide) were formulated as handmade edible gelatins and given three days per week for 17 months to 18 dogs (DRF, dogs receiving fungi); a second group was maintained without fungi (CD, control dogs). All individuals were dewormed at months 0, 3, 7, 10 and 13, and it was observed that the levels of helminths egg-output were reduced by 96–98% fourteen days after each treatment. Fecal egg counts of STHs were similar in both groups until the 6th–8th months, and then remained significantly lower in DRF than in CD (42–100% ascarids; 30–100% trichurids and ancylostomatids). According to the results, and considering that gelatin treats have always been fully accepted, it is concluded that this new formulation offers an efficient solution to decrease the risk of infection among dogs maintained in shelters, and is therefore recommended.

## 1. Introduction

Canine shelters provide a community service consisting of admitting and caring for lost or abandoned dogs, which are provided appropriate veterinary attention comprising physical examination and deworming. A quarantine period is observed before they are placed into plots with more dogs; females are neutered to avoid overpopulation and facilitate their adoption. Prevalence levels of digestive endoparasites ranging from 17% to 98% have been reported in canine shelters in different countries, mostly caused by protozoa and helminths [1,2,3,4,5]. Ascarids, trichurids, and ancylostomatids are very frequently detected soil-transmitted helminths (STHs), with a direct cycle, and their transmission is enhanced in dirt floors which favor the development of eggs passed through the feces of parasitized dogs to their infective stages after two to six weeks, thus increasing the risk of new infections [6]. Even though dogs left at shelters are expected to be housed temporarily until a new owner is found, this does not happen as regularly as desired, and therefore, recently admitted individuals sharing the kennels with others previously housed by private owners are at risk of exposure to infective stages which develop in soil [7].

Ascarids or ancylostomatids are potentially zoonotic agents, and their control among pets in shelters is also very important to reduce the risk for keepers who may become infected [3,5,6,7,8], or of zoonotic transmission when these animals are adopted by families. This reinforces the need to preclude development of infective stages in the soil, through routine cleaning of the kennels and removing of feces, together with canine deworming among the measures necessary [9].

For the purpose of reducing the presence of infective stages of certain endoparasites, one interesting procedure relies on the use of certain soil-filamentous saprophytic fungi. More specifically, *Pochonia chlamydosporia* and *Mucor circinelloides* are able to penetrate the eggs of *Toxocara canis*, *Baylisascaris procyonis*, and *Trichuris vulpis* and destroy the inner embryo; *Duddingtonia flagrans* and *Monacrosporium thaumasium* elaborate traps in their mycelium for catching larvae developed from eggs of strongyles in the soil [10,11,12]. There is scarce information on the practical application of these fungi against gastrointestinal nematodes affecting dogs [13]; it has been reported the viability of eggs of *T. canis*, *T. leonina*, and *T. vulpis* was halved in the feces of puppies given dry feed previously sprayed with a blend of chlamydospores with complementary parasiticide activity, including *M. circinelloides* (ovicide) and *D. flagrans* (larvicide) [14]. The main goal in the current investigation was to analyze the usefulness of a new edible formulation, consisting of gelatin with a mixture of *M. circinelloides* and *D. flagrans*, to limit the survival of STHs’ infective stages in the soil and, therefore, reduce infection among dogs housed in a shelter.

## 2. Materials and Methods

### 2.1. Animal Shelter

**Figure 1:**
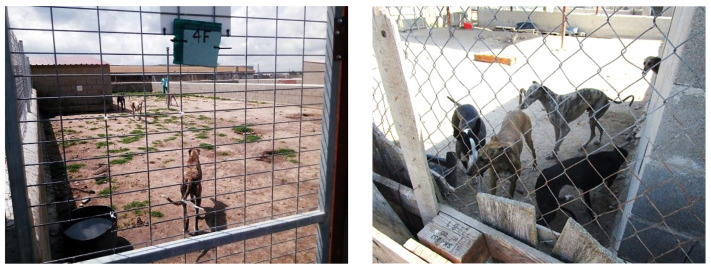
The “Scooby” shelter is the largest in Spain, and dogs are maintained in paddocks with dirt floors, which facilitates the development of soil-transmitted helminths from eggs to their infective stages.

“Scooby” (Medina del Campo, Valladolid) (41°18′48″ N, 4°53′23″ W) is the largest shelter in Spain, housing more than 600 cats and dogs together with 200 farm animals (horses, cattle, sheep, donkeys) (Figure 1). There is a strip of land of about 20 m between dogs and cats and the other livestock. About 80% of the rescued dogs are hounds including greyhounds for hunting wild leporidae (rabbits and hares), which explains why the highest intensity in the constant flow of incoming dogs occurs around early February, the end of the hunting season. With the aim of promoting their adoption, agreements have been established with other European countries including Belgium, the Netherlands, France, and the United Kingdom.

Dogs are housed in kennels with floors of dirt and cement, wire-fenced, provided with a refuge area (built of concrete and wood) where they can shelter from bad weather (Figure 1). Water is available ad libitum in drinkers, and feeding consists of dry feedstuff and bread scraps (by private donation). The enclosures are cleaned almost every day, by removing the feces manually in the morning, and high-pressure water is applied periodically to the refuge areas.

### 2.2. Control of Parasites

All newly incorporated animals receive an antiparasitic treatment and are then maintained under quarantine for one week before placement in plots with other dogs. Parasiticide treatment consists of a single dose of Helm-ex^®^ (Laboratorios Karizoo, Barcelona, Spain) chewable tablets composed of febantel (525 mg), pyrantel emboate (504 mg), and praziquantel (175 mg), active against all relevant cestodes and nematodes. Administration is carried out according to the body weight of the dog (up to 5 kg, half a tablet; from 5 to 10 kg, one tablet; from 10 to 15 kg, one and a half; from 15 to 20 kg, two tablets; from 20 to 25 kg, two and a half; and above 25 to 30 kg, three tablets per animal). This deworming schedule is repeated approximately every 3–4 months.

### 2.3. Elaboration of Edible Treats with Fungal Chlamydospores

In the present investigation, two filamentous fungi with proven parasiticide activity were used: *Mucor circinelloides* (CECT 20824; ovicide) and *Duddingtonia flagrans* (CECT 20823; larvicide). These species were isolated from soil and fecal samples of livestock and wild captive animals, then deposited in the Spanish Type Culture Collection (CECT, Valencia, Spain).

For the purpose of obtaining chlamydospores of both simultaneously, fungi were cultured in a submerged medium (COPFr) for 1.5–2 months at RT [14]. These chlamydospores were formulated as gelatin treats (additional information cannot be provided due to these foods pending registration), prepared in the lab by mixing edible gelatin powder, honey, and liquid medium containing 5–7.5 × 10^3^ chlamydospores of both *M. circinelloides* and *D. flagrans*/mL. Once completely homogenized, the blend was heated under microwave for a brief period and placed into silicone molds (approximately 40 mL/each), quenched at 4–6 °C to enhance gelation and then at −35 °C until frozen. Finally, the products were lyophilized and packed into reusable plastic bags. This formulation ensures a dosage of 2–3 × 10^5^ chlamydospores of each parasiticide fungus is provided to each dog.

### 2.4. Study Design

The experimental design was approved by the Ethical Committee of the University of Santiago de Compostela (Spain; protocol number CTM2015-65954b) and complied with the Directive 2010/63/EU. A total of six kennels with six adult mix-breed dogs in each were utilized in the current study, and two groups formed by three kennels each (18 dogs) were considered:

- CD (control dogs): dogs receiving anthelmintic treatment periodically (a single dose of Helm-ex^®^ as previously described) and one gelatin without chlamydospores three times a week (every Tuesday, Thursday and Saturday) for 17 months.
- DRF (dogs receiving fungi): dogs dewormed as in CD. One gelatin containing chlamydospores of the parasiticide fungi was given to each individual, three times a week (every Tuesday, Thursday, and Saturday) for 17 months.

### 2.5. Evaluation of the Control Measures against Soil-Transmitted Helminths (STHs)

Faced with the difficulty of taking fecal samples from the rectum of each individual, feces were collected directly from the ground in plastic sample beakers with covers. Every month for a period of 17 months, members of the COPAR Research Group (University of Santiago de Compostela, Spain) went to Scooby and collected a total of 18 fecal samples from each kennel. The time elapsed between collection and analysis was ca. 12 h, and in the meantime, samples were kept refrigerated.

In order to evaluate the initial status of canine infection by parasites, as well as the efficacy of the measures adopted during the trial (conventional deworming and biological control with spores of parasiticide fungi), feces were examined in duplicate using the McMaster technique with saturated saline solution (ρ = 1.2 g/dL) [14]. Briefly, three grams of each sample were weighed and placed in a bottle, then emulsified in 42 mL of water and shaken vigorously until completely broken down. This emulsion was filtered through a 150 µm pore diameter sieve and passed into two 15 mL glass tubes. After centrifugation at 1500 rpm for 10 min, the sediment was homogenized in saturated saline solution and observed in a McMaster chamber under an optical microscope (Leica DM2500) at 10×. Results were expressed as the numbers of eggs per gram of feces (EPG).

The efficacy of the anthelmintic was estimated fourteen days after each administration, based on the FECR (fecal egg count reduction), and efficacy was considered when FECR ≥ 95% [14].

The kinetics of the parasite eggs were evaluated monthly, and the ratios between the values of CD and DRF were estimated. By calculating the FECR values throughout the trial, two comparative risk periods were determined: a non-risky feces period (NRFP) when FECR = 100% (=eggs of STHs were not observed), and low-risk feces period (LRFP) if FECR > 90% and <100% (=fecal counts of STHs eggs reduced by one tenth).

### 2.6. Acceptance of Edible Formulations with Fungal Spores and Analysis of Harmful Effects

During the study, the ingestion of gelatin treats by the dogs in the two groups was checked. To confirm the absence of possible adverse effects of fungal spores, all the dogs were examined regularly for weakness, and changes in appetite, thirst, or consistency of feces. Attention was paid also to the respiratory function and to the possibility of emergence of skin damage (redness, blistering, peeling, or cracking) or hair loss.

### 2.7. Statistical Analysis

According to the Kolmogorov–Smirnov test, data were not normally distributed (Z values < 0.05), and the Levene’s test showed the variances were not homogeneous (*p* < 0.05). The non-parametric Mann–Whitney U test was performed at a significance level of *p* < 0.05. All tests were carried out using the statistical package SPSS, version 20 (IBM SPSS Inc., Chicago, IL, USA).

## 3. Results

Eggs of STHs (soil-transmitted helminths) found in the feces of dogs were identified as ascarids (*Toxocara canis*, *Toxascaris leonina*), trichurids (*Trichuris vulpis*), and ancylostomatids (*Ancylostoma caninum*). Oocysts of *Cystoisospora canis* and cysts of *Giardia* sp. were seldom detected, and these data were not considered in the current research.

### 3.1. Efficacy of Deworming

Dogs in the study received a total of five anthelmintic treatments (Table 1). An elevated efficacy was recorded against all STHs, with average values of 98% vs. *T. canis*, 97% vs. *T. leonina*, 96% vs. *T. vulpis*, and 98% against *A. caninum* in CD, and of 98%, 96%, 97% and 98%, respectively, in DRF. No significant differences were demonstrated among the two groups.

### 3.2. Kinetics of STHs Fecal Egg-Output

**Figure 2:**
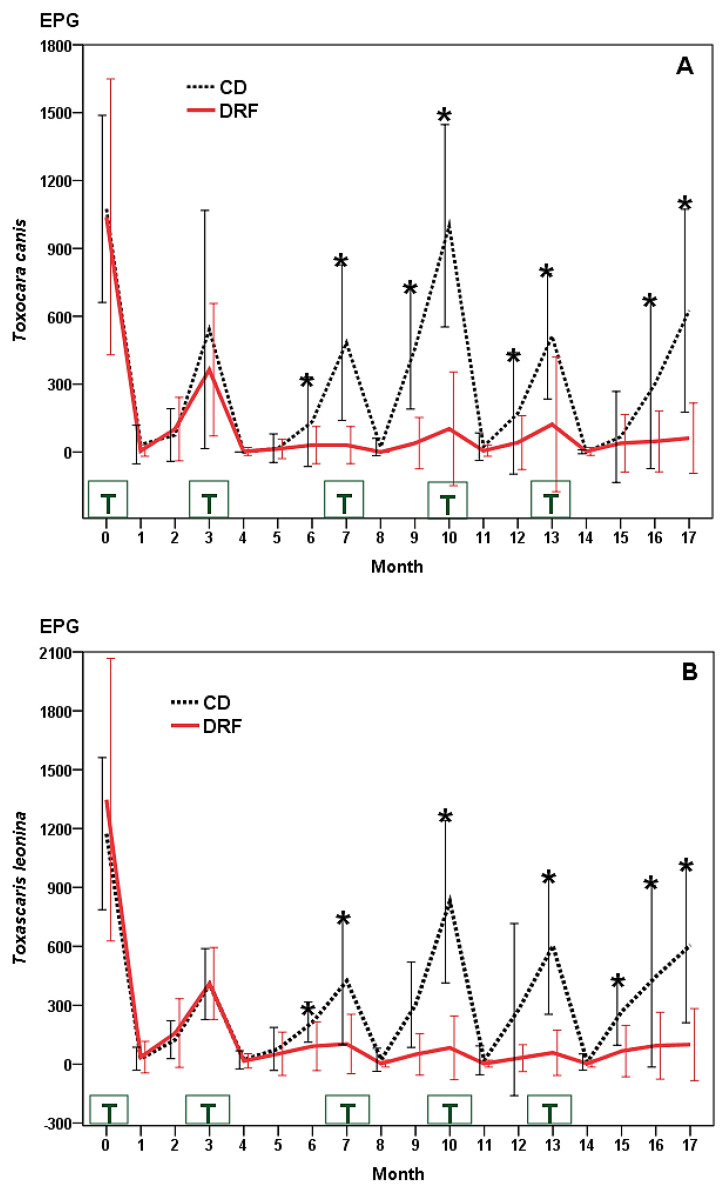
Dynamics of eggs of *T. canis* (**A**) and *T. leonina* (**B**) in dogs from the “Scooby” shelter (Spain). CD: control dogs (not receiving fungi); DRF: dogs provided (three times a week) with chlamydospores of *M. circinelloides* and *D. flagrans*. T: anthelmintic treatment. (*): statistical differences.

Numbers of eggs of *T. canis* and *T. leonina* around 1100 EPG were observed in the two groups of dogs at the beginning of the study (Figure 2A,B), increased after the first deworming until the 3rd month (near to 300 EPG), then deworming was administered again. From this point, numbers of *T. canis* and *T. leonina* eggs between 500 and 1000 were achieved in the controls (CD), and counts lower than 125 EPG in DRF until the end of study, representing a 42–100% diminution (Z = −8.649, *p* = 0.001 *T. canis*, and Z = −8.926, *p* = 0.001 *T. leonina*).

**Figure 3:**
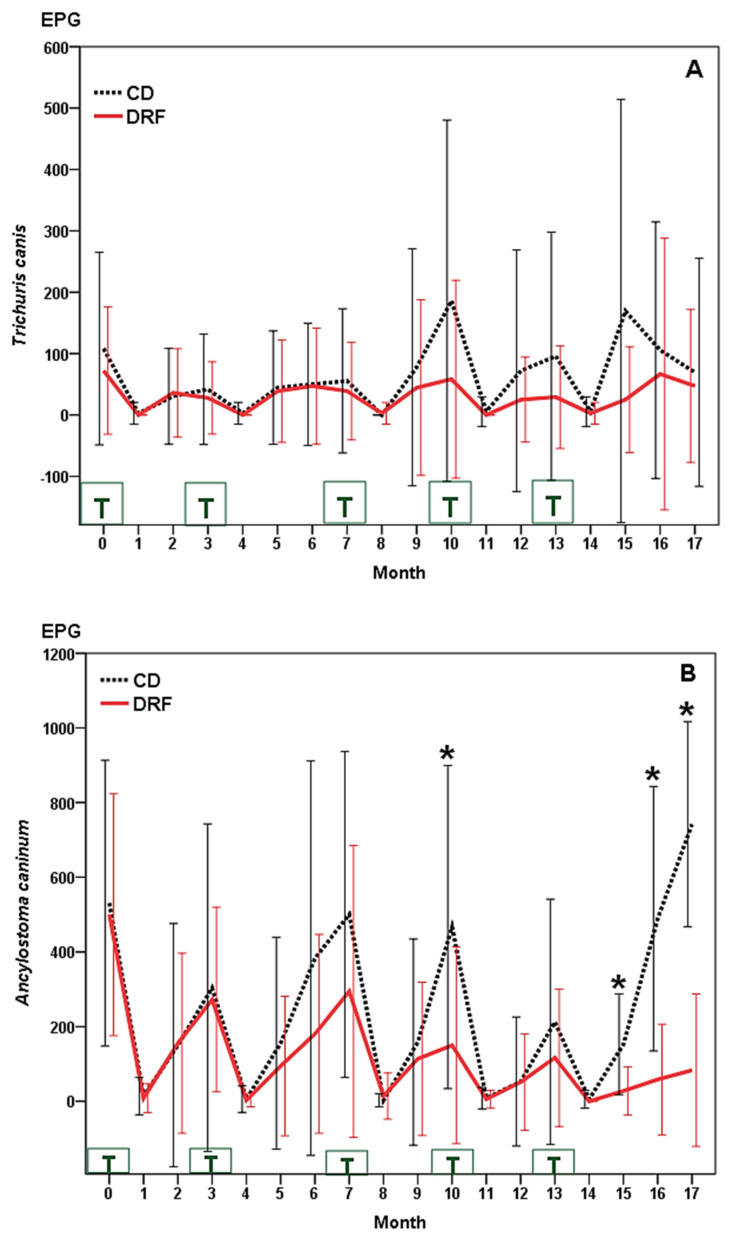
Dynamics of eggs of *T. vulpis* (**A**) and *A. caninum* (**B**) in dogs from the “Scooby” shelter (Spain). CD: control dogs (not receiving fungi); DRF: dogs provided (three times a week) with chlamydospores of *M. circinelloides* and *D. flagrans*. T: anthelmintic treatment. (*): statistical differences.

During the first 8 months of study, the dynamics of eggs of T. vulpis in both groups of dogs were analogous (Figure 3A), with values ranging from 0 to 108 EPG. In CD, numbers around 170 EPG (months 10 and 15) were recorded, with counts below 70 EPG in DRF (reduced by 30–100%) (Z = −3.242, *p* = 0.002).

The egg-count values of *A. caninum* were comparable in the two groups of dogs until the 6th month (Figure 3B). Thereafter, different peaks around 450 and 750 EPG were observed in CD, whereas a significant reduction was obtained in DRF, especially from the 9th month, with values < 100 EPG (33–100% lower) (Z = −5.070, *p* = 0.001).

### 3.3. Effect of the Integrated Control Strategy

The ratios between the EPG values of each STHs in DRF and CD were estimated, in order to analyze the effect of the integrated control strategy (Table 2). At the beginning of the study, values close to 1 for all the STHs were found, but DRF/CD ratios between 0.6 and 0.1 were obtained for *T. canis* and *T. leonina* from the 6th month of study. The ratios for *T. vulpis* ranged from 0.57 to 0 from the 9th month of study, and ratios between 0.55 and 0 were attained from the 13th month for *A. caninum*.

No differences were observed regarding the NRFP (non-risky feces period), for which a period of one month was observed for *T. canis*, *T. leonina*, and *A. caninum* both in CD and DRF (Table 1). The LRFP (low-risk feces period) was extended two–three times in the DRF after the first deworming, in comparison with the CD, and intervals between two and four months were obtained for *T. canis*, *T. leonina* and *T.vulpis*, and one–two months for *A. caninum*.

Table 3 summarizes the numbers of dogs positive according to coprological analysis throughout the study. All the individuals in the controls (100%) passed eggs of ascarids two–three months after every deworming, 40–60% were positive for *T. vulpis* and 72–100% for *A. caninum*. In the dogs receiving the chlamydospores, it was noted that the prevalence of dogs positive for *T. canis* decreased from the 5th month of study, and levels around 27–40% were obtained until the end of the study period. Similar results were attained from the 9th month of study for *T. leonina*, with values about 39–44%. The percentages of dogs taking the chlamydospores and passing eggs of *T. vulpis* ranged between 21 and 39%, while values lower than 56% were not observed for *A. caninum* until the 15th month.

### 3.4. Level of Acceptance of Gelatin and Analysis of Adverse Effects

None of the dogs refused to take the gelatin treats. No problems were observed regarding the appearance of digestive, respiratory, or cutaneous disorders.

## 4. Discussion

In the current study, eggs of STHs (*T. canis*, *T. leonina*, *T. vulpis* and *A. caninum*) were identified in the feces of two groups of dogs kept in a shelter, then anthelmintic treatment was successfully administered [14]. Nevertheless, elevated counts of eggs were observed in the feces two–three months later, which indicates that they became infected due to the soil contaminated by infective stages of the helminths [15,16]. Consequently, deworming was required every three–four months, and a total of five anthelmintic treatments were administered during a 17-months period. Certain hygiene procedures such as regular removal of feces, washing, and brushing, are often recommended and practiced in canine shelters every one–two days to avoid pathogens originating from the feces attaining their infective stages [6,17]. Based on the reduction in viability of helminth eggs in feces obtained by providing puppies with chlamydospores of two fungi with parasiticide activity (*M. circinelloides* and *D. flagrans*) [14], in the present research one group of efficiently dewormed dogs was given a new edible formulation of this blend of chlamydospores consisting of dried handmade gelatin-based treats, administered three times a week. This formulation was apparently palatable and tasty for dogs. During the first six months, no differences were observed between the two groups, but STH egg-counts reduced significantly towards the end of the study in the feces of dogs taking the treats with the chlamydospores, and ratios lower than 0.6 in respect to the controls were recorded, representing a reduction ≥ 40%. Previous studies reported in vitro antagonism of certain filamentous fungi such as *Purpureocilium lilacinus*, *P. chlamydosporia*, *Trichoderma* sp., or *M. circinelloides* against eggs of ascarids [18,19], supported by the ability to delay or interfere with their development (ovistatic effect) and to destroy the inner embryo ovicidal effect). Viability of eggs of *Ascaris suum* dropped by 50% and 66% when the filamentous fungi *Clonostachys rosea* or *Trichoderma atrobrunneum* were sprayed on feces of pigs, while the effect on the eggs of *Lemurostrongylus* sp. was 25% and 33%, respectively [20]. Data obtained in the current research suggest that chlamydospores formulated as dried gelatin-based treats reached the feces of dogs, developed to hyphae, and decreased the survival of eggs passed in the feces, and their possibility of evolving to infective stages [21], therefore limiting the risk of infection in these dogs.

Canine shelters play an essential role in caring for animals abandoned by their owners, or lost, or otherwise unable return home. Regarding the control of their health status, special emphasis is put on certain infections caused by parasites including protozoa or helminths, through the quarantine and deworming of newly arrived animals [8]. It has been stated that dogs receiving a single deworming at the moment of their introduction into shelters might not be considered parasite-free, and a new test should be carried out before introducing them into a kennel, in order to avoid soil contamination [22], although this is very hard to apply mainly due to economic reasons. The situation worsens in larger kennels with extensive land or sand for dogs can enjoy and socialize, where these conditions enhance the risk of polyparasitism by several STHs [21,23].

Bearing in mind that in the current investigation, dogs became infected one–two months after successful deworming, it appears necessary to observe useful strategies complementary to deworming for successful control of STHs in canine shelters, while several points should be considered. Firstly, an interval between two to six weeks is needed for eggs to attain their infective stages, and eggs of ascarids or trichurids present a highly protective eggshell enabling elevated resistance to unfavorable conditions, and thus can remain infective for long periods (months to years), especially in moist and shaded areas [6,24,25,26,27]. Secondly, disinfectant products frequently applied in kennels, veterinary clinics, and households against *T. canis* fail to eliminate the risk of infection, because of their inability to affect embryogenesis and viability [28]. Thirdly, isolates of *A. caninum* with multiple anthelmintic resistance have been recently reported in the USA, presumably related to deworming in racing greyhound kennels [29].

Contamination of ground by soil-transmitted helminths depends on infected hosts passing eggs in feces, and on the ability of the organisms to attain infective stages and to survive. Another very important factor is the period elapsing between deworming and the reappearance of parasites in feces, when the chance of soil contamination might be significantly increased over short time intervals [30]. For this purpose, in the present study non-risky feces periods (NRFP) were defined as when fecal analyses were negative (FECR = 100%), and low-risk feces periods (LRFP) if fecal egg-counts of STHs were reduced by one tenth (FECR values between 90% and 100%). No differences were observed regarding the NRFP for *T. canis*, *T. leonina*, and *A. caninum*, but the LFRP extended two–three times in the group given the fungi, which supports the hypothesis that the viability and evolution of eggs in their feces are strongly limited, therefore reducing the hazard of soil contamination.

The prevalence of dogs reinfected after deworming appears a very interesting topic due to the possibility of elevated percentages of animals passing low quantities of eggs in their feces also contributing to increased soil contamination levels. In the current investigation, differences according to the prevalence of dogs infected after the administration of anthelmintic were not observed until the 6th month of study, hence it is inferred that the administration of fungal spores did not seem to affect the infective stages that had already developed in the soil. However, the reduction of infection levels in the dogs provided with chlamydospores, together with the values of STH egg-outputs from this point (6th month), was attributable to the diminishing risk of reinfection, because of the decrease of viability and development rates of the eggs passed in their feces [14].

## 5. Conclusions

To reduce the risk of infection with certain helminths affecting dogs cared for in a shelter, the formulation of chlamydospores of *M. circinelloides* and *D. flagrans* as gelatin treats offers a useful solution that is easy to use and without additional work for keepers. The drying of the treats facilitates their easy preservation at room temperature. The treatment provides an effective solution with a sustainable approach to decrease the frequency of deworming in those dogs, and is therefore strongly recommended.

## Figures and Tables

**Table 1:** Efficacy of deworming on dogs cared for in “Scooby” shelter (Spain).

	Soil-Transmitted Helminths (STHs)											
Deworming number	***Toxocara canis***						***Toxascaris leonina***					
	FECR (95% CI)		NRFP (months)		LRFP (months)		FECR (95% CI)		NRFP (months)		LRFP (months)	
	CD	DRF	CD	DRF	CD	DRF	CD	DRF	CD	DRF	CD	DRF
1	97 (96, 98)	99 (94, 100)	0	0	2	2	98 (97, 99)	97 (93, 100)	0	0	1	0
2	100	99 (94, 100)	1	0	2	4	95 (92, 97)	96 (93, 99)	0	0	2	3
3	95 (94, 97)	100	0	1	1	3	95 (93, 97)	97 (93, 100)	1	0	2	3
4	98 (97, 99)	95 (91, 99)	0	0	1	2	98 (97, 99)	97 (92, 100)	0	0	2	3
5	100	98 (92, 100)	1	0	2	4	98 (97, 99)	95 (92, 99)	0	0	1	4
	***Trichuris vulpis***						***Ancylostoma caninum***					
	FECR (95% CI)				ERP		FECR (95% CI)				ERP	
	CD	DRF	CD	DRF	CD	DRF	CD	DRF	CD	DRF	CD	DRF
1	97 (94, 100)	100	0	1	3	3	97 (96, 99)	98 (95,100)	0	0	1	1
2	93 (89, 100)	100	0	1	4	4	98 (96,100)	99 (95, 100)	0	0	1	2
3	100	93 (90, 97)	0	0	2	3	99 (98, 100)	95 (93, 98)	0	0	1	1
4	97 (94, 99)	100	0	1	3	3	98 (97, 99)	96 (92, 100)	0	0	2	2
5	94 (89,99)	90 (85, 95)	0	0	1	4	97 (94, 99)	100	0	1	1	4

CD: dogs dewormed at 0, 3, 7, 10 and 13 months; DRF: dogs dewormed at 0, 3, 7, 10 and 13 months and given chlamydospores of *M. circinelloides* and *D. flagrans*. LRFP: Low-risk feces period (90% < FECR < 100%); NRFP: Non-risky feces period (FECR = 100%).

**Table 2:** Relationship between the values for helminth egg-output in two groups of dogs housed in the “Scooby” shelter (Spain).

Month of Study	MAD	Ratio between the EPG Values in DRF and CD			
		* Toxocara canis *	* Toxascaris leonina *	* Trichuris vulpis *	* Ancylostoma caninum *
0 (**T**)	0	0.97	1.15	0.67	0.94
1	1	0.17	1.30	0.00	0.60
2	2	1.35	1.27	1.18	1.04
3 (**T**)	3	0.67	1.01	0.67	0.90
4	1	-	0.75	0.00	0.50
5	2	0.83	0.68	0.88	0.61
6	3	0.23	0.43	0.94	0.47
7 (**T**)	4	0.06	0.24	0.70	0.59
8	1	0.00	0.13	-	5.00
9	2	0.08	0.17	0.57	0.72
10 (**T**)	3	0.10	0.10	0.31	0.32
11	1	0.24	0.14	0.00	0.67
12	2	0.24	0.11	0.35	0.97
13 (**T**)	3	0.24	0.10	0.30	0.55
14	1	0.60	0.25	0.50	0.00
15	2	0.58	0.25	0.15	0.18
16	3	0.16	0.21	0.63	0.12
17	4	0.10	0.17	0.68	0.11

CD: control dogs (not receiving fungi); DRF: dogs provided (three times a week) with chlamydospores of *M. circinelloides* and *D. flagrans*. T: anthelmintic treatment. MAD: month after deworming.

**Table 3:** Numbers of dogs which tested positive for the presence of STHs in feces.

Month of Study	Deworming Number	MAD	*Toxocara canis*		*Toxascaris leonina*		*Trichuris vulpis*		*Ancylostoma caninum*	
			CD	DRF	CD	DRF	CD	DRF	CD	DRF
**0**	**1**	0	18/18	18/18	18/18	18/18	13/18	13/18	18/18	18/18
1		1	6/18	2/18	7/18	7/18	1/18	1/18	3/18	2/18
2		2	12/18	11/18	18/18	13/18	6/18	8/18	7/18	11/18
3	2	3	18/18	18/18	18/18	18/18	7/18	7/18	13/18	14/18
4		1	1/18	1/18	7/18	6/18	1/18	1/18	1/18	1/18
5		2	3/18	4/18	14/18	7/18 *	7/18	7/18	8/18	7/18
6		3	15/18	5/18 *	18/18	12/18	7/18	7/18	12/18	10/18
7	3	4	18/18	5/18 *	18/18	12/18	7/18	7/18	16/18	13/18
8		1	3/18	2/18	5/18	1/18	1/18	1/18	1/18	2/18
9		2	18/18	4/18 *	18/18	7/18 *	6/18	5/18	10/18	10/18
10	4	3	18/18	6/18 *	18/18	8/18 *	9/18	5/18	18/18	11/18 *
11		1	3/18	2/18	3/18	1/18	2/18	2/18	3/18	2/18
12		2	10/18	5/18	10/18	7/18	11/18	5/18	4/18	7/18
13	5	3	18/18	7/18 *	18/18	8/18 *	11/18	5/18	14/18	11/18
14		1	1/18	1/18	3/18	1/18	2/18	1/18	2/18	2/18
15		2	5/18	4/18	18/18	8/18 *	8/18	4/18	18/18	6/18 *
16		3	15/18	5/18 *	14/18	8/18	13/18	6/18 *	18/18	6/18
17		4	16/18	6/18 *	18/18	8/18 *	11/18	7/18	18/18	7/18 *

CD: control dogs (not receiving fungi); DRF: dogs provided (three times a week) chlamydospores of *M. circinelloides* and *D. flagrans*. Statistical differences are indicated by an asterisk (*). MAD: month after deworming.

## Data Availability

The data presented in this study are available on request from the corresponding author. The data are not publicly available due to research group policy.
